# Clinic, Home, and Kiosk Blood Pressure Measurements for Diagnosing Hypertension: a Randomized Diagnostic Study

**DOI:** 10.1007/s11606-022-07400-z

**Published:** 2022-03-03

**Authors:** Beverly B Green, Melissa L Anderson, Andrea J Cook, Kelly Ehrlich, Yoshio N Hall, Clarissa Hsu, Dwayne Joseph, Predrag Klasnja, Karen L Margolis, Jennifer B McClure, Sean A Munson, Mathew J Thompson

**Affiliations:** 1 Kaiser Permanente Washington Health Research Institute, Seattle, WA USA; 2 Washington Permanente Medical Group, Seattle, WA USA; 3 Kidney Research Institute, University of Washington Department of Medicine, Seattle, WA USA; 4 University of Michigan, School of Information, Ann Arbor, MI USA; 5 HealthPartners Institute, Minneapolis, MN USA; 6 Department of Human Centered Design and Engineering, University of Washington, Seattle, WA USA; 7 Department of Family Medicine, University of Washington, Seattle, WA USA

**Keywords:** hypertension, diagnosis, blood pressure determination, blood pressure monitoring, ambulatory, blood pressure monitoring, home

## Abstract

**Background:**

The US Preventive Services Task Force recommends blood pressure (BP) measurements using 24-h ambulatory monitoring (ABPM) or home BP monitoring before making a new hypertension diagnosis.

**Objective:**

Compare clinic-, home-, and kiosk-based BP measurement to ABPM for diagnosing hypertension.

**Design, Setting, and Participants:**

Diagnostic study in 12 Washington State primary care centers, with participants aged 18–85 years without diagnosed hypertension or prescribed antihypertensive medications, with elevated BP in clinic.

**Interventions:**

Randomization into one of three diagnostic regimens: (1) clinic (usual care follow-up BPs); (2) home (duplicate BPs twice daily for 5 days); or (3) kiosk (triplicate BPs on 3 days). All participants completed ABPM at 3 weeks.

**Main Measures:**

Primary outcome was difference between ABPM daytime and clinic, home, and kiosk mean systolic BP. Differences in diastolic BP, sensitivity, and specificity were secondary outcomes.

**Key Results:**

Five hundred ten participants (mean age 58.7 years, 80.2% white) with 434 (85.1%) included in primary analyses. Compared to daytime ABPM, adjusted mean differences in systolic BP were clinic (−4.7mmHg [95% confidence interval −7.3, −2.2]; *P*<.001); home (−0.1mmHg [−1.6, 1.5];*P*=.92); and kiosk (9.5mmHg [7.5, 11.6];*P*<.001). Differences for diastolic BP were clinic (−7.2mmHg [−8.8, −5.5]; *P*<.001); home (−0.4mmHg [−1.4, 0.7];*P*=.52); and kiosk (5.0mmHg [3.8, 6.2]; *P*<.001). Sensitivities for clinic, home, and kiosk compared to ABPM were 31.1% (95% confidence interval, 22.9, 40.6), 82.2% (73.8, 88.4), and 96.0% (90.0, 98.5), and specificities 79.5% (64.0, 89.4), 53.3% (38.9, 67.2), and 28.2% (16.4, 44.1), respectively.

**Limitations:**

Single health care organization and limited race/ethnicity representation.

**Conclusions:**

Compared to ABPM, mean BP was significantly lower for clinic, significantly higher for kiosk, and without significant differences for home. Clinic BP measurements had low sensitivity for detecting hypertension. Findings support utility of home BP monitoring for making a new diagnosis of hypertension.

**Trial Registration:**

ClinicalTrials.gov NCT03130257 https://clinicaltrials.gov/ct2/show/NCT03130257

**Supplementary Information:**

The online version contains supplementary material available at 10.1007/s11606-022-07400-z.

## INTRODUCTION

Elevated blood pressure (BP) is the leading contributor to cardiovascular events and mortality.^1, 2^ While most adults with hypertension take antihypertensive medications, many are unaware they have high BP. A study using National Health and Nutrition Examination Survey 2017–2018 data estimated that 23% of US adults with high BP (systolic ≥140 mmHg or diastolic ≥90 mmHg) were unaware they had hypertension and were untreated.^3^

For patients with high screening BP in clinic, US Preventive Services Task Force (USPSTF)^4^ and other hypertension guidelines recommend follow-up BP testing outside of clinics by 24-h ambulatory BP monitoring (ABPM) or home BP monitoring (HBPM), to avoid overdiagnosis and unnecessary treatment.^5–7^

Currently, ABPM is infrequent in the USA, partly from lack of availability, low reimbursement, and perceptions of lower patient acceptability.^8^ HBPM is an alternative, but patients need to use validated monitors, be trained on proper use, and take multiple measurements,^9–11^ leading to questions about the accuracy of using HBPM to diagnose hypertension. BP kiosks in pharmacies or clinic waiting areas are an alternative for HBPM, but to our knowledge, no studies have compared kiosk to ABPM for making a new hypertension diagnosis. Blood Pressure Checks for Diagnosing Hypertension (BP-CHECK) was a randomized diagnostic study comparing the accuracy of clinic BP, HBPM, and kiosk BPs to ABPM for making a new diagnosis of hypertension.

## METHODS

### Setting and Population

The setting was 12 KPWA primary care centers in Western Washington. Enrollment was May 11, 2017, to March 4, 2019. Study design and methods were published.^12^ Electronic health records (EHRs) were used to identify individuals aged 18–85 with elevated BP (≥138 mmHg systolic or ≥88 mmHg diastolic at last outpatient visit) with no hypertension diagnosis in the prior 2 years and no prescribed antihypertensive medications in the prior 12 months. EHR data were used to exclude patients with BP ≥180 mmHg systolic or ≥110 mmHg diastolic; pregnancy; life-limiting illness (e.g., end-stage renal failure); and conditions that might make participation difficult (e.g., dementia) or BP monitoring less accurate (e.g., atrial fibrillation).

Potentially eligible individuals were mailed an invitation with a study description and $2 bill and called to confirm eligibility. Those willing to participate were scheduled for screening visits. At visits, a research assistant confirmed individuals had not engaged in heavy exercise, used tobacco, or had caffeinated drinks in the prior 30 min. Individuals sat in a chair with back support and the upper left arm was measured with an appropriately sized cuff used.^11^ After 5 minutes' rest, BP was measured twice 1 min apart using a validated Omron 907XL monitor.^13^

Individuals with BP ≥140 mmHg systolic or ≥90 mm Hg diastolic on both measurements were eligible. Those with mean ≥180 mmHg systolic or ≥110 mm Hg diastolic were excluded and told to make a physician’s appointment.

### Randomization and Blinding

The biostatistician used R statistical software (version 3.2.2) to randomly assign patients to clinic, home, or kiosk groups. Randomization was stratified by clinic, age (<60 or ≥60 years), and mean baseline systolic BP (<150 or ≥150 mmHg), with random block sizes of 3 or 6 within each stratum. Except for study biostatisticians, investigators were blinded until all outcome data were collected. Participants and study staff were aware of group assignments.

### Diagnostic Tests

The clinic group received routine care for high BP at KPWA. Participants were instructed to make an appointment at their clinic for a BP recheck. Clinic BP measurements are usually taken by a medical assistant using a wall-mounted aneroid BP monitor (Welch Allyn Tyco)^14^ or, less frequently, with a validated oscillometric BP monitor (GE CARESCAPE V100).^15^ For high initial BP (systolic BP ≥140 mmHg or diastolic BP ≥90 mmHg), standard procedure is to measure BP again after at least 5 min.^16^ If BP is elevated again, the patient’s clinician is notified to make a plan and a follow-up BP-check visit is scheduled. These steps are repeated until BP is <140/90 mmHg.

Home participants received a validated Bluetooth-enabled oscillometric Omron N786 BP monitor^17^ with an appropriately sized upper-arm cuff to take duplicate measurements twice daily for 5 days: after rising and at bedtime (total 20 measurements).^18^ Home BPs were collected by study staff via Bluetooth.

Kiosk participants were asked to measure BP using validated PharmaSmart BP kiosks^19^ at KPWA clinics or nearby pharmacies, with triplicate measurements on three separate days.^20^ Measurements were linked to participants via a kiosk smartcard and collected using the vendor’s cloud-based service.

Clinic, home, and kiosk participants received verbal and written instructions. All participants received the same reminder 2 weeks after their initial visit to complete their diagnostic assignment if they had not already done so.

### Reference Standard

All participants were asked to return at 3 weeks for ABPM diagnostic testing by validated Welch Allyn 7100 ABPM (cuff size based on arm circumference, non-dominant arm),^21^ measuring BP every 30 min, 7AM to 11PM, and hourly, 11PM to 7AM. Patient participants and their physicians received ABPM results and were advised to follow up on tests that were positive for hypertension. ABPM results and communications were documented in the EHR (home and kiosk BPs were not).

### Outcomes

The primary outcome was participant’s mean systolic BP for assigned diagnostic method using all available BP data. Mean diastolic BP and diagnostic accuracy were secondary outcomes. Daytime ABPM was defined as the mean of BPs collected 7AM-11PM. Nighttime plus daytime measurements were used for secondary outcomes of mean 24-h and mean nighttime ABPM. Adverse events potentially related to study participation were reported using the question: “Since your last research visit, have you experienced any new or serious health concerns?” BP outcomes at 6 months included receipt of a new hypertension diagnosis in the EHR (based on new ICD-10 code I-11, I-12, or 1-13). Prespecified subgroup comparisons were based on potential to influence diagnostic performance: baseline systolic BP (<150 mmHg vs. ≥150); age (<60 vs. ≥60 years); arm size (<33 vs. ≥33 cm); body mass index (BMI as kg/m^2^; <30 vs. ≥30); cardiovascular disease (CVD) risk; and race.

### Sample Size

With a sample size of 510 (170 per group) and assuming an outcome ascertainment rate of at least 80%, we could detect a 4.1-mmHg difference in systolic and a 2.8-mmHg difference in diastolic BP between any two groups (assuming standard deviation 12.1 mmHg systolic and 8.3 mmHg diastolic). BP differences and standard deviation were based on prior studies comparing clinic, home, and kiosk BP to ABPM.^18, 20, 22^

### Analyses

Primary and secondary outcomes were analyzed among participants completing ≥1 BP diagnostic test measurement and ≥14 daytime reference test ABPM measurements.^23^ Group differences in the comparability of the diagnostic test systolic BP relative to ABPM (primary outcome), were assessed using linear regression models with the dependent variable defined as within-person difference in systolic BP between the mean diagnostic test and ABPM measures. Models included indicators for diagnostic group, with adjustment for age, sex, BMI, education, and baseline systolic and diastolic BP. Generalized estimating equations with robust standard error were used to relax normality assumptions.^24^ Fisher protected least significant difference approach was used to protect against multiple comparisons, requiring that the omnibus test of any differences between groups was statistically significant before making pairwise comparisons.^25^ To address bias due to missing data, a sensitivity analysis was conducted using inverse probability weighting for the primary analysis population only. Group differences for diastolic BP measurement (secondary outcome) were assessed using similar methods. In exploratory analyses, we restricted analyses to those defined a priori as adherent to assigned diagnostic regimen, based on evidence for home and kiosk,^18, 20^ and usual care for clinic: clinic, 1 outpatient clinic BP; home, 16 measurements over ≥4 days; kiosk, 6 measurements over ≥2 days. Effect modification by baseline BP and participant characteristics (e.g., age, race) was assessed by including interactions between diagnostic method and subgroups. Bland-Altman plots were used to show agreement between diagnostic BP measurements and ABPM.

Secondary analyses assessed the diagnostic performance of clinic, home, and kiosk BP measures by estimating the sensitivity and specificity of each diagnostic method compared to the reference standard, daytime ABPM. Hypertension diagnosis according to the reference standard was defined as mean daytime ABPM systolic ≥135 mmHg and/or diastolic ≥85 mmHg. For each diagnostic method, we defined a positive test as mean diagnostic BP above a threshold for hypertension of systolic BP ≥140 mmHg and/or diastolic BP ≥90 mmHg for clinic and systolic ≥135 mmHg and/or diastolic ≥85 mmHg for home and kiosk.^26, 27^ Sensitivity was estimated by fitting a logistic regression model with an indicator for a positive test result as the dependent variable and diagnostic group as the dependent variable among participants with hypertension by ABPM. Specificity estimates were similar but with an indicator for a negative test result as dependent variable and diagnostic group as independent variable among participants without hypertension by ABPM. We also estimated positive and negative predictive values, and positive and negative likelihood ratios for each group. Exploratory analyses estimated these diagnostic metrics for a variety of systolic/diastolic pairs of thresholds for defining a positive diagnostic test using receiver operating characteristic (ROC) curves and computed area under the curve (AUC) separately for systolic and diastolic BP. AUC was not calculated for the curve based on systolic/diastolic pairs, because there is no way to vary the paired thresholds across the range of possible values.

Additional exploratory analyses included diagnostic performance assessment using the following: (a) mean 24-h ABPM instead of daytime ABPM as the reference standard with hypertension thresholds of mean systolic ≥130 mmHg and mean diastolic ≥80 mmHg and (b) American College of Cardiology/American Heart Association (ACC/AHA) recommendations for diagnosing stage 1 hypertension (≥130 mmHg systolic or ≥80 mmHg diastolic for mean daytime ABPM, clinic, home, and kiosk BP).^5, 26^ Analyses used Stata statistical software (release 15). Statistical inference used 2-sided hypothesis tests, and significance threshold *P<.*05.

## RESULTS

**Figure 1:**
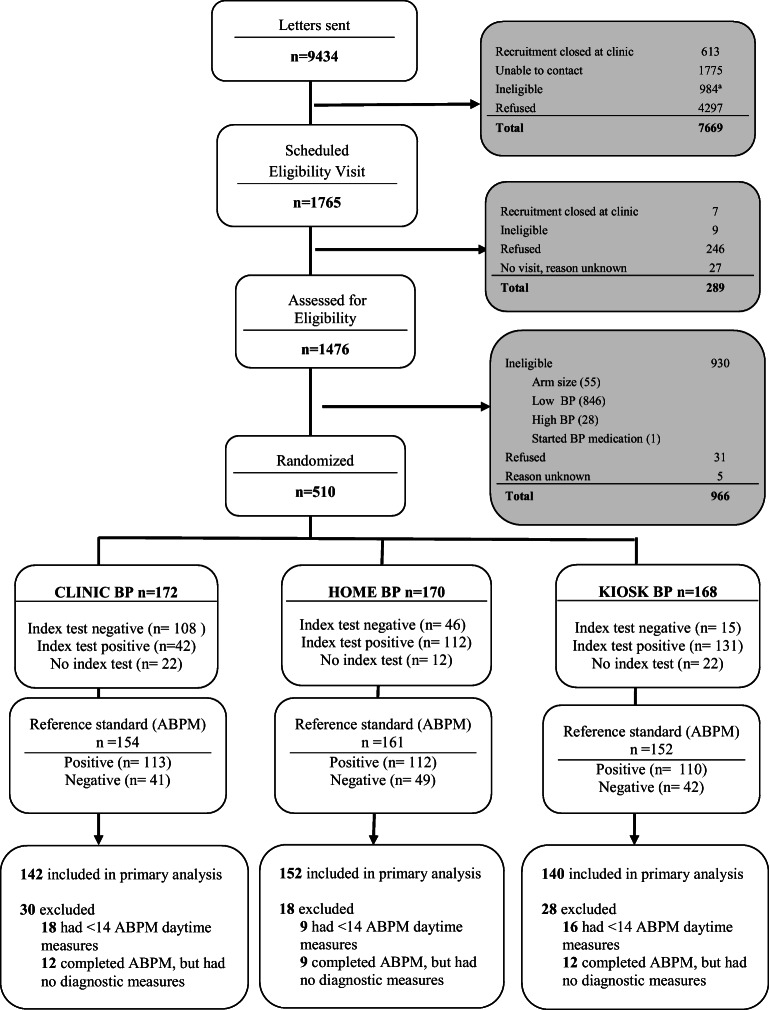
Recruitment, randomization, and follow-up in the BP-CHECK Study. The most common reasons for ineligibility on the follow-up call after invitation letters were sent were going out of town (>2 weeks in the next 2 months), *n*=509; leaving the health plan, *n*=174; and unable to converse in English, *n*=174. Abbreviations: BP, blood pressure; ABPM, 24-h ambulatory blood pressure monitoring.

We mailed 9434 invitation letters to patients with EHR data showing BP ≥138/88 at their last clinic visit, no hypertension diagnosis, and no antihypertensive medications (**Fig.** **1**). Ineligibility was most commonly because BPs were not elevated at the screening visit. Patient characteristics were similar across randomization groups, with 48% female, 80% white, mean age 59 years, and mean baseline BP 150/88 mmHg (**Table** **1**). Among those randomized to clinic, home, and kiosk, 142 (82.6%), 152 (89.4%), and 140 (83.3%) had sufficient BP data for primary analyses (**Fig.** **1****,** Appendix Table 1). ABPM adherence was similar across groups with 91.6% overall completion.

**Table 1:** Baseline Characteristics of Study Participants by Randomization Group*

	**Clinic BP**	**Home BP**	**Kiosk BP**
Number of participants	*N*=172	*N*=170	*N*=168
Age, mean in years (SD)	58.1 (14.2)	59.3 (13.2)	58.9 (12.9)
Sex, *n* (%)			
Female	82 (47.7)	86 (50.6)	79 (47.0)
Race, *n* (%)†			
White	136 (79.1)	137 (80.6)	136 (81.0)
African American	13 (7.6)	13 (7.7)	8 (4.8)
Asian	9 (5.2)	10 (5.9)	9 (5.4)
Other race‡	14 (8.1)	10 (5.9)	15 (8.9)
Hispanic, *n* (%)	8 (4.8)	3 (1.8)	8 (4.9)
Education, *n* (%)			
High school or less	20 (11.9)	16 (9.5)	16 (9.6)
Some college	44 (26.2)	48 (28.6)	51 (30.7)
College graduate	52 (31.0)	50 (29.8)	48 (28.9)
Graduate degree	52 (31.0)	54 (32.1)	51 (30.7)
BMI, mean (SD)	31.3 (6.9)	30.4 (6.7)	28.7 (4.9)
Diabetes, *n* (%)	4 (2.3)	5 (2.9)	4 (2.4)
Upper left arm circumference, cm, mean (SD)§	32.9 (4.3)	32.5 (3.8)	32.0 (4.1)
Cardiovascular disease, *n* (%)	1 (0.6)	1 (0.6)	1 (0.6)
Moderate-to-high risk for cardiovascular disease (%)^II^	91 (53.9)	92 (54.8)	88 (53.7)
Baseline systolic BP mmHg, mean (SD)¶	149.4 (9.8)	149.9 (11.0)	150.1 (9.4)
Baseline diastolic BP mmHg, mean (SD)¶	88.5 (9.3)	87.9 (9.2)	88.0 (9.6)

^*^Missing: Hispanic (*n*=9), education (*n*=8), BMI (*n*=8), diabetes (*n*=2), CVD (*n*=2), CVD risk (*n*=9)

^†^The eligibility phone survey collected race using a categorical race question, with an “other” option. If the question was not answered, categorical race data from the electronic health record were used.

^‡^Other race: kiosk (American Indian/Alaskan Native=2; multi-racial=6; other self-reported categories=7); home (Pacific Islander/Native Hawaiian=2; multi-racial=4; other self-reported categories=4); clinic (American Indian/Alaska Native=1; multi-racial=6, other self-reported categories=7)

^§^Upper left arm circumference at the midpoint between the olecranon process and acromion prominence

^II^Moderate-to-high risk for CVD was defined as age ≥75, statin prescribed in the past 12 months, or ≥15% 10-year risk using Framingham equations ^43, 44^. When cholesterol laboratory data were unavailable, BMI was used to calculate CVD risk ^45, 46^

^¶^Baseline BP measures are the mean of two measurements at the baseline study visit

Abbreviations: *BMI*, body mass index (weight in kilograms divided by height in meters squared); *BP*, blood pressure; *CVD*, cardiovascular disease; *SD*, standard deviation; *mmHg*, millimeter of mercury

### Primary and Secondary Outcomes

Compared to mean daytime ABPM, adjusted mean differences in systolic BP were as follows: clinic (−4.7 mmHg [95% confidence interval −7.3, −2.2]; *P*<.001); home (−0.1 mmHg [−1.6, 1.5]; *P*=.92); and kiosk (9.5 mmHg [7.5, 11.6]; *P*<.001) (**Table** **2**). Differences for diastolic BP were as follows: clinic (−7.2 mmHg [−8.8, −5.5]; *P*<.001); home (−0.4 mmHg [−1.4, 0.7]; *P*=.52); and kiosk (5.0 mmHg [3.8, 6.2]; *P*<.001). Bland-Altman plots demonstrate the variability of within-person differences (Appendix Figure 1).

**Table 2:** Differences in Mean Systolic and Diastolic Diagnostic BP and Mean Daytime ABPM by Randomization Group*

		**Daytime** **ABPM**	**Diagnostic protocol**	**Adjusted† mean difference** **(diagnostic-ABPM)**	
	*N*	Mean (SD)	Mean (SD)	Mean difference (95% CI)	*P*-value‡
**Systolic BP**					
Clinic	142	138.7 (11.8)	133.9 (13.5)	−4.7 (−7.3, −2.2)	<0.001
Home	152	137.2 (10.7)	137.1 (11.5)	−0.1 (−1.6, 1.5)	0.92
Kiosk	140	137.4 (11.5)	147.1 (14.0)	9.5 (7.5, 11.6)	<0.001
**Diastolic BP**					
Clinic	142	86.0 (8.9)	79.2 (8.3)	−7.2 (−8.8, −5.5)	<0.001
Home	152	86.0 (8.5)	85.8 (8.2)	−0.4 (−1.4, 0.7)	0.52
Kiosk	140	86.8 (9.9)	91.2 (10.3)	5.0 (3.8, 6.2)	<0.001

^*^Among individuals who had ≥1 diagnostic (clinic, home, kiosk) measurement, and ≥14 daytime ABPM measurements

+Linear regression models adjusted for age, sex, body mass index, education, and baseline systolic and diastolic BP (from screening visit), estimated with generalized estimating equations with robust (sandwich) variance estimation

^‡^*P*-value for mean difference between each method and ABPM. We also tested the difference between groups for the outcome of mean difference between diagnostic and ABPM measures. Differences in systolic BP were significant at *P*<.001 for clinic vs. home and home vs. kiosk, and *P*.002 for clinic vs. home. Adjusted mean difference in diastolic BP between groups was significant at *P*<.001 for clinic vs. home, clinic vs. kiosk, and home vs. kiosk.

Abbreviations: *ABPM*, ambulatory blood pressure monitoring; *SD*, standard deviation; *CI*, confidence interval; *BP*, blood pressure

Using a diagnostic threshold of daytime mean ABPM ≥135 mmHg systolic or ≥85 mmHg diastolic, 71.7% of participants tested positive for hypertension. Sensitivities for detecting hypertension were 31.1% (95% CI 22.9, 40.6) clinic, 82.2% (73.8, 88.4) home, and 96.0% (90.0, 98.5) kiosk. Specificities were 79.5% (64.0, 89.4) clinic, 53.3% (38.9, 67.2) home, and 28.2% (16.4, 44.1) kiosk (**Table** **3**). False positive rates were 5.6% clinic, 13.8% home, and 20.0% kiosk.

**Table 3:** Diagnostic Performance of Clinic, Home, and Kiosk BP Monitoring Compared to Mean Daytime ABPM*^,†^

	**Clinic**	**Home**	**Kiosk**
	*N*=142	*N*=152	*N*=140
Reference test (ABPM) positive for hypertension, %^‡^	72.5	70.4	72.1
Sensitivity, % (95% CI)	31.1 (22.9, 40.6)	82.2 (73.8, 88.4)	96.0 (90.0, 98.5)
Specificity, % (95% CI)	79.5 (64.0, 89.4)	53.3 (38.9, 67.2)	28.2 (16.4, 44.1)
Positive predictive value, % (95% CI)	80.0 (64.8, 89.7)	80.7 (72.2, 87.1)	77.6 (69.5, 84.1)
Negative predictive value, % (95% CI)	30.4 (22.3, 40.0)	55.8 (40.9, 69.8)	73.3 (46.7, 89.6)
Positive likelihood ratio (95% CI)	1.51 (0.77, 2.99)	1.76 (1.27, 2.44)	1.34 (1.09, 1.63)
Negative likelihood ratio (95% CI)	0.87 (0.71, 1.07)	0.33 (0.20, 0.54)	0.14 (0.05, 0.41)
True positive (hypertension), *n* (%)	32 (22.5)	88 (57.9)	97 (69.3)
True negative (normotensive), *n* (%)	31 (21.8)	24 (15.8)	11 (7.9)
False positive, *n* (%)	8 (5.6)	21 (13.8)	28 (20.0)
False negative (missed hypertension), *n* (%)	71 (50.0)	19 (12.5)	4 (2.9)
Correctly classified, %	44.4	73.7	77.1

^*^Among individuals who had ≥1 diagnostic (clinic, home, kiosk) measurement, and ≥14 daytime ABPM measurements.

^†^Diagnostic thresholds were ≥140 mmHg systolic and/or ≥90 mmHg diastolic for clinic and ≥135 and/or ≥85 mmHg for home, kiosk, and ABPM

^‡^Prevalence of hypertension based on ABPM was 71.7% across all groups combined

Abbreviations: *BP*, blood pressure; *ABPM*, ambulatory BP monitoring; *CI*, confidence interval; *mmHg*, millimeter of mercury

**Figure 2:**
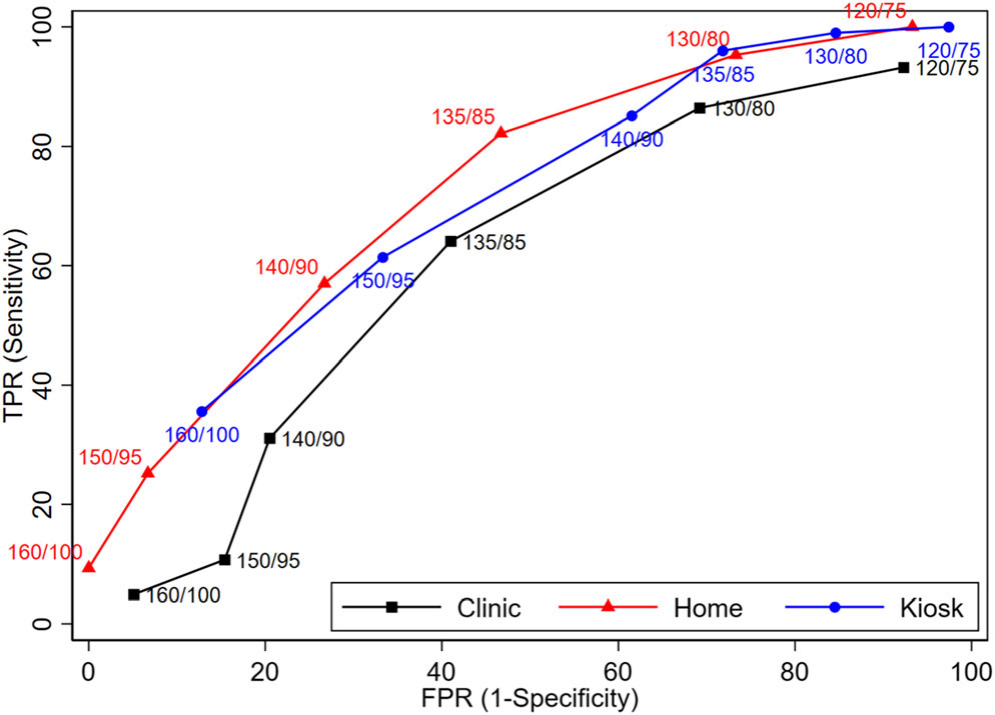
Receiver operating characteristic curves based on paired systolic/diastolic thresholds. TPR, true positive rate; FPR, false positive rate.

Area under the curve (AUC) analyses for systolic BP (Appendix Figure 2) suggested that HBPM (AUC 0.77, CI 0.69, 0.84) performed better than clinic (AUC 0.64, CI 0.54, 0.71) and similar to kiosk (AUC 0.75, CI 0.70, 0.84) for identifying elevated systolic BP compared to ABPM. For diastolic BP, kiosk (AUC 0.86, CI 0.79, 0.92) performed better than both clinic (AUC 0.75, CI 0.67, 0.83) and HBPM (AUC 0.75, CI 0.67, 0.92). However, caution should be used in interpreting these results as hypertension diagnosis is based on either a high systolic BP or high diastolic BP and not each value separately. Using a variety of thresholds for receiver operating characteristic (ROC) curves demonstrated that home and kiosk performed better than clinic over a range of BP thresholds (**Fig.** **2**). While diagnostic accuracy was similar for home and kiosk, thresholds needed to achieve similar sensitivity and specificity were higher for clinic.

### Sensitivity and Exploratory Analyses

Planned sensitivity analyses used inverse probability weighting to account for missing outcome data (Appendix Table 2), or were limited to participants adherent to protocol based on a prespecified number of clinic, home, or kiosk BP measurements (Appendix Table 3). Results did not differ from the main analyses. Using ABPM 24-h mean BP with diagnostic threshold ≥130 mmHg systolic or ≥80 mmHg (Appendix Table 4), or ABPM nighttime BP (Appendix Table 5) with diagnostic threshold ≥120 mmHg systolic or ≥70 mmHg made little difference in clinic, home, and kiosk diagnostic performance. Using ACC/AHA thresholds for stage 1 hypertension (systolic BP ≥130 mmHg or diastolic BP ≥80 mmHg) increased hypertension prevalence to 86.2% and improved the sensitivity of clinic, home, and kiosk, but at the expense of specificity (Appendix Table 6). Very high Clinic BPs (≥160/100 mmHg, specificity 94.9%) provided some assurance that white coat hypertension could be ruled out (Appendix Table 7).

### Subgroup Analyses

Differences between mean daytime ABPM and mean clinic, home, and kiosk systolic and diastolic BP and diagnostic performance varied little by patient characteristics (Appendix Table A8.a-c).

### Intermediate Outcomes

Among the 71.7% (335/467) of individuals with hypertension based on ABPM testing (reference standard), 40.9% (137/335) had a hypertension diagnosis recorded in the EHR by their provider between the baseline and 6-month follow-up visit, with no differences by randomization group.

### Adverse Events

No adverse events related to clinic, home, or kiosk BP monitoring were reported. ABPM-related adverse events were reported by 36 participants with 39 complaints: arm discomfort (*n*=20), skin irritation (*n*=7), sleep disturbance (*n*=7), and anxiety or restlessness (*n*=5).

## DISCUSSION

In a real-world diagnostic study conducted in primary care, home mean systolic and diastolic BP did not differ significantly from ABPM. In contrast, clinic BPs were significantly lower and kiosk BPs were significantly higher than ABPM.

A systematic review for the USPSTF evaluated the accuracy of office-based BPs compared to ABPM for screening and confirming hypertension.^6, 7^ Most but not all studies found that office-based BPs were higher than ABPM.^6, 7^ Most studies used automated oscillometric or manual mercury BP monitors with attention to optimal technique and multiple measurements averaged. In our study, BP measurements were mainly by medical assistants as part of usual care, typically using aneroid syphyngomanometers. Possible explanations for our finding of clinic BP lower than ABPM include improper measurement technique (e.g., not inflating cuff above true systolic BP, deflating cuff too rapidly, difficulties discerning Korotkoff sounds), end-number rounding, and unintentional bias toward lower BP readings.^28, 29^ Our findings align with a cluster randomized trial that found systolic BP 7.5 mmHg lower in clinics using manual measurements than clinics with BP measured by validated oscillometric automated monitors (*P*<.001), with substantial reductions in end-number rounding (systolic BP manual 71%, versus automated 18%; *P*<.001).^30^ Routine use of automated monitors, especially with multiple readings taken over several minutes, could lead to average clinic BPs closer to ABPM;^31, 32^ however, this practice is uncommon in the USA.^33^

Another reason that clinic BPs may have been lower than ABPM in our setting is because BP is rechecked only if the initial BP was >140/90 as recommended by our healthcare organization and national guidelines.^34^ This policy, recommended by our healthcare organization and national guidelines was informed in part by Handler et al., which analyzed data from National Health and Nutrition Examination Surveys among 22,641 adults with 3 BP readings.^16^ Among those with Joint National Committee 7 (JNC7)^35^ defined stage 1 hypertension (systolic BP ≥140 to <160 mmHg or diastolic ≥90 to <100 mmHg) or stage 2 hypertension (systolic ≥160 mmHg or diastolic ≥100 mmHg), 18.2% and 33.5%, respectively, were reclassified to a lower stage using the mean of the second and third BP readings, and less than 0.5% were reclassified to a higher stage. This was interpreted to mean that no additional measures are needed if the first BP is below a threshold, but if the first BP is high, it should be checked again after 5 min of rest. Criticisms of this paper include lack of comparison to ABPM or repeated BPs taken on separate days. Thus, downward reclassification after repeated BP measurements may lead to a bias of using the lowest BP instead of the true average, which our study suggests.

Lower clinic BPs resulted in lower sensitivity for detecting hypertension than home and kiosk. In the USPSTF review,^6, 7^ initial screening BPs had low sensitivity and moderate specificity for detecting ABPM-confirmed hypertension, with pooled sensitivity and specificity from 15 studies of 54% and 90%, respectively. However, confirmatory office-based BPs (i.e., after a high screening BP) tended to overdiagnose hypertension with pooled sensitivity and specificity from 8 studies of 80% and 55%.^6, 7^ These results are in sharp contrast to our study, with clinic sensitivity 31.1% and specificity 79.5%. The negative likelihood ratio (calculated from both sensitivity and specificity) was close to 1.0, indicating that a clinic BP less than 140 mmHg systolic and 90 mmHg diastolic (negative test) had little impact on the likelihood that the ABPM would be negative. Our study suggests that confirmatory BPs in clinic, as practiced in routine care, may be more likely to underdiagnose than overdiagnose hypertension.

Home BP was similar to ABPM. BP is highly variable, particularly among individuals with untreated hypertension, with systolic often varying by 30–40 mmHg or more during daytime hours.^36^ Home provided many more BPs, with average standard deviation smaller than single or duplicate clinic measurements. Home had higher sensitivity than clinic for detecting hypertension, but at the expense of specificity. However, as most participants had hypertension, false positive rates were relatively low. Our Home BP results are similar to those reported by the USPSTF review,^6, 7^ which had HBPM pooled sensitivity and specificity 84.0% and 60.0%, respectively. While ABPM is considered the “gold standard,” controversy remains.^27^ Both ABPM and HBPM are more predictive of cardiovascular events than clinic BPs. Three studies demonstrated that ABPM more closely correlates with left ventricular hypertrophy/mass than HBPM.^37–39^ However, no trials have compared the effectiveness of ABPM and HBPM in preventing CVD events.

Mean systolic and diastolic BPs were significantly higher for kiosk than ABPM. Kiosk diagnostic accuracy was still better than clinic across a range of BP thresholds. The BP kiosk we used was validated against mercury manometers.^19^ Another study compared it to ABPM,^20^ with 100 individuals without treated hypertension taking triplicate kiosk BP measurements at local pharmacies on 4 separate days. Kiosk BP was only slightly higher than ABPM (by 2.3±9.5 mmHg systolic, 2.2±6.9 mmHg diastolic). Kiosks in our study were often in busy waiting rooms, possibly leading to higher BPs. While participants were instructed to rest 5 min before measurements, we are not certain they did. Additional study could determine if protocol or equipment adjustments (e.g., rest period, quiet location) improve BP kiosk performance.

Our study used ACC/AHA stage 2 recommendations for making a new diagnosis of hypertension^5^. Using ACC/AHA stage 1 BP thresholds, 86% of our population tested positive for hypertension. The ACC/AHA guidelines recommend lifestyle behavior change to lower BP for individuals with stage 1 hypertension at low risk for CVD and antihypertensive medications for those at moderate-to-high CVD risk. Most of our population was at moderate-to-high CVD risk and would qualify for antihypertensive medications at the lower stage 1 threshold.^5, 40^

Even though home BP monitoring performed better than clinic and kiosk, many implementation challenges remain. The tools we used to systematically collect and average home BPs (Bluetooth connectivity and a database for averaging BPs) are not typically available in primary care. Furthermore, although participants and their physicians received ABPM results and the diagnostic interpretation of the test, only 41% of those with a positive test had a hypertension diagnosis recorded in the EHR by 6 months. We know of only one implementation study that is testing different strategies for improving hypertension diagnosis, with results not yet published.^41^ This US study is testing a multilevel strategy that includes provider presentations, patient information, nurse training for teaching patients to conduct HBPM, EHR-embedded clinical decision support, and increased access to a culturally adapted ABPM service. Results of this study might inform next steps for improving hypertension diagnosis.

### Limitations

Study limitations were first, since the study was conducted at a single healthcare organization, results might differ at other settings with different standards for measuring BP, such as use of automated BP. Second, African American and other racial/ethnicity groups were underrepresented. Third, although the accuracy of all BP monitors used was validated by established protocols, results might differ with other monitors.^42^ Fourth, participants were aware of their diagnostic group assignment and that they had high BP. Contextual and behavioral factors might have influenced BP diagnostic results. Fifth, diagnostic performance may have differed if we included individuals with lower BPs. Sixth, statistical comparisons of AUCs for ROCs of systolic or diastolic BP between diagnostic methods and the reference standard should be interpreted cautiously because the diagnosis of hypertension is based on both systolic or diastolic BP, rather than considering each measure separately, and thresholds for diagnosing hypertension differ across guidelines and subgroups. Last, assessment of comparative performance across measurement methods might have been more robust if participants had completed all three diagnostic regimens. However, our study compared hypertension diagnosis methods as used in real-world settings, which would not be possible with that study design.

## CONCLUSIONS

In this diagnostic study, compared to ABPM, clinic had significantly lower and kiosk significantly higher mean BP measures. HBPM was not significantly different, lending further credibility to the utility of home measurements. Most participants with high BP on screening and ABPM diagnostic testing did not receive a hypertension diagnosis. New strategies are needed to improve hypertension diagnosis.

## Supplementary Information

ESM 1 (DOCX 448 kb)
