# Supplementary material for: Clinic, Home, and Kiosk Blood Pressure Measurements for Diagnosing Hypertension: a Randomized Diagnostic Study
Source: J Gen Intern Med. 2022 Mar 3;37(12):2948–56. doi: 10.1007/s11606-022-07400-z (PMC9485334; doi:10.1007/s11606-022-07400-z)
Supplement: Supplementary file 1 — (DOCX 448 kb) [file 11606_2022_7400_MOESM1_ESM.docx]

**Appendix Table 1. BPs Collected During the Diagnostic Period (Between Visit 1 and Visit 3)^*^ by Randomization Group**

|  | **Clinic BP**  **N=172** | **Home BP**  **N=170** | **Kiosk BP**  **N=168** |
| --- | --- | --- | --- |
| **Clinic, Home, or Kiosk BP measurement** |  |  |  |
| No BP data, n (%) | 22 (12.8) | 12 (7.1) | 22 (13.1) |
| Adherent to the group assignment protocol, n (%)^†^; 95% Confidence interval | 150 (87.2)  (81.3, 91.8) | 154 (90.6)  (85.2, 94.5) | 114 (67.9)  (60.2, 74.8)^‡^ |
| Group assignment diagnostic BP measurements^*,†,§^ |  |  |  |
| Number attained, mean (SD) | 1.4 (0.5) | 27.6 (15.4) | 9.5 (2.9) |
| Number attained, range | 1 to 3^II^ | 16 to 94 | 6 to 26 |
| **ABPM** |  |  |  |
| No ABPM, n, (%) | 18 (10.5) | 7 (4.1) | 14 (8.3) |
| Some ABPM data, (<14 daytime BP), n (%) | 0 (0.0) | 2 (1.2) | 2 (1.2) |
| Adherent to ABPM protocol (≥14 daytime BP), n (%)^¶^ | 154 (89.5) | 161 (94.7) | 152 (90.5) |
| Daytime BPs^¶^ |  |  |  |
| Number attained, mean (SD) | 26.8 (3.5) | 26.6 (3.1) | 27.4 (3.4) |
| Number attained, range | 15 to 43 | 15 to 41 | 15 to 41 |
| 24-hour BPs (all) ^¶^ |  |  |  |
| Number attained, mean (SD) | 36.3 (4.0) | 36.1 (3.7) | 37.1 (3.9) |
| Number attained, range | 22 to 53 | 25 to 53 | 25 to 61 |
| Nighttime ^¶^ |  |  |  |
| Number attained, mean (SD) | 9.5 (1.1) | 9.5 (1.1) | 9.7 (1.0) |
| Number attained, range | 2 to 12 | 2 to 12 | 3 to 15 |
| **Analytic samples** |  |  |  |
| Included in the primary analysis^**^, n (%) | 142 (82.6) | 152 (89.4) | 140 (83.3) |
| Included in the per-protocol assessments^††^, n (%) | 142 (82.6) | 149 (87.7) | 111 (66.1) |

^*^ Collected between Visit 1 (baseline visit) and Visit 3 (the visit to return the ABPM device)

^†^ Adherence to group-assigned diagnostic protocol included BP measures collected between Visit 1 (baseline visit) and Visit 2 (the visit to put on the ABPM device) and defined as having: at least 1 clinic BP recorded in the EHR; at least 16 home BPs completed over at least 4 separate days, and at least 6 kiosk BPs completed over at least 2 separate days

^‡^ Kiosk vs. Home, *P*<.001; Kiosk vs. Clinic, *P*<.001

^§^ Among individuals adherent to group assignment protocol

^II^ Of the 150 participants with at least 1 clinic BP measurement, 141, had measurements at a single visit, and 9 had measurements at 2 separate visits (on 2 separate days). Of the 159 person-visits with BP measurement, 114, 44, and 1 visits had 1, 2, and 3 BP measurements respectively

Adherence to ABPM protocol required ≥14 daytime BPs with no requirement on nighttime measurements

^¶^ Among individuals adherent to ABPM protocol

^**^ Individuals were included in the primary analyses if they had ≥14 daytime BPs (by ABPM), and at least one diagnostic BP measurement (Kiosk, Home, or Clinic, according to their randomization group)

^††^ Individuals were included in the per-protocol analyses if they had ≥14 daytime BPs (by ABPM) and were adherent to group assignment BP data collection between Visit 1 and Visit 2.

Abbreviations: BP, blood pressure; ABPM, ambulatory BP monitoring; EHR, electronic health record; SD, standard deviation; N, number for group; n, number for subgroup

**Appendix Table 2.** **Sensitivity Analysis Using Inverse Probability Weighting^*^**

|  |  | **Daytime**  **ABPM** | **Diagnostic protocol** | **Adjusted^†^ mean difference**  **(diagnostic-ABPM)** | |
| --- | --- | --- | --- | --- | --- |
|  | N | Mean (sd) | Mean (sd) | Mean difference  (95% CI) | *P*-value^‡^ |
| **Systolic BP** |  |  |  |  |  |
| Clinic | 142 | 138.7 (11.8) | 133.9 (13.5) | -5.3 (-7.8, -2.8) | <0.001 |
| Home | 152 | 137.2 (10.7) | 137.1 (11.5) | 0.0 (-1.6, 1.5) | 0.96 |
| Kiosk | 140 | 137.4 (11.5) | 147.1 (14.0) | 9.7 (7.7, 11.8) | <0.001 |
| **Diastolic BP** |  |  |  |  |  |
| Clinic | 142 | 86.0 (8.9) | 79.2 (8.3) | -7.1 (-8.7, -5.5) | <0.001 |
| Home | 152 | 86.0 (8.5) | 85.8 (8.2) | -0.2 (-1.3, 0.9) | 0.70 |
| Kiosk | 140 | 86.8 (9.9) | 91.2 (10.3) | 4.5 (3.3, 5.7) | <0.001 |

^*^ Including all participants who were included in the primary analysis and using inverse probability weighting to make their characteristics more similar to the full randomized population. Logistic regression models used to estimate model weights included age, sex, randomization group, baseline systolic and diastolic BP, education, smoking status, body mass index, and interactions between randomization group and baseline systolic and diastolic BP

^†^ Linear regression outcome models adjusted for age, sex, body mass index, education, and baseline systolic and diastolic BP (from screening visit), estimated with generalized estimating equations with robust (sandwich) variance estimation

^‡^ *P*-value for mean difference between each method and ABPM

Abbreviations: BP, blood pressure; ABPM, ambulatory BP monitoring; SD, standard deviation; CI, confidence interval

**Appendix Table 3 Sensitivity Analysis Using Participants Adherent to Clinic, Home, and Kiosk BP Monitoring Protocols***

|  |  | **Daytime**  **ABPM** | **Diagnostic protocol** | **Adjusted^†^ mean difference**  **(diagnostic-ABPM)** | |
| --- | --- | --- | --- | --- | --- |
|  | N | Mean mmHg (sd) | Mean mmHg (sd) | Mean difference mmHg  (95% CI) | *P*-value^‡^ |
| **Systolic BP** |  |  |  |  |  |
| Clinic | 142 | 138.7 (11.8) | 133.9 (13.5) | -4.8 (-7.3, -2.2) | <0.001 |
| Home | 149 | 137.0 (10.7) | 136.9 (11.4) | -0.1 (-1.7, 1.4) | 0.87 |
| Kiosk | 111 | 137.1 (10.9) | 146.2 (13.1) | 9.0 (6.9, 11.1) | <0.001 |
| **Diastolic BP** |  |  |  |  |  |
| Clinic | 142 | 86.0 (8.9) | 79.2 (8.3) | -7.2 (-8.8, -5.6) | <0.001 |
| Home | 149 | 85.9 (8.4) | 85.5 (8.0) | -0.5 (-1.5, 0.6) | 0.40 |
| Kiosk | 111 | 87.0 (9.1) | 91.0 (10.0) | 4.6 (3.2, 5.9) | <0.001 |

**^*^** Adherence to group-assigned diagnostic protocol included BP measures collected between Visit 1 (baseline visit) and Visit 2 (the visit to put on the ABPM device) and defined as having: at least 1 clinic BP recorded in the EHR; at least 16 home BPs completed over at least 4 separate days, and at least 6 kiosk BPs completed over at least 2 separate days

^†^ Linear regression models adjusted for age, sex, body mass index, education, and baseline systolic and diastolic BP (from screening visit), estimated with generalized estimating equations with robust (sandwich) variance estimation

^‡^ *P*-value for mean difference between each method and ABPM

Abbreviations: BP, blood pressure; ABPM, ambulatory BP monitoring; SD, standard deviation; CI, confidence interval; mmHg, millimeter of mercury

**Appendix Table 4. Diagnostic Performance of Clinic, Home, and Kiosk BP Monitoring Compared**

**to Mean 24-hour ABPM^*, †^**

|  | **Clinic** | **Home** | **Kiosk** |
| --- | --- | --- | --- |
|  | N=141 | N=151 | N=138 |
| Prevalence of Hypertension Based on ABPM^‡^ | 81.6 | 78.8 | 77.5 |
| Sensitivity (95% CI) | 30.4 (22.7, 39.4) | 82.4 (74.4, 88.2) | 94.4 (88.1, 97.5) |
| Specificity (95% CI) | 80.8 (61.3, 91.8) | 68.8 (51.0, 82.3) | 29.0 (15.9, 47.0) |
| Positive Predictive Value (95% CI) | 87.5 (73.3, 94.7) | 90.7 (83.6, 94.9) | 82.1 (74.3, 87.9) |
| Negative Predictive Value (95% CI) | 20.8 (14.0, 29.8) | 51.2 (36.6, 65.6) | 60.0 (34.8, 80.8) |
| Positive Likelihood Ratio (95% CI) | 1.58 (0.69, 3.65) | 2.64 (1.57, 4.44) | 1.33 (1.06, 1.67) |
| Negative Likelihood Ratio (95% CI) | 0.86 (0.69, 1.08) | 0.26 (0.16, 0.40) | 0.19 (0.07, 0.50) |
| True Positive (Hypertension), n (%) | 35 (24.8) | 98 (64.9) | 101 (73.2) |
| True Negative (Normotensive), n (%) | 21 (14.9) | 22 (14.6) | 9 (6.5) |
| False Positive, n (%) | 5 (3.6) | 10 (6.6) | 22 (15.9) |
| False Negative (Missed Hypertension), n (%) | 80 (56.7) | 21 (13.9) | 6 (4.4) |
| Correctly Classified (%) | 39.7 | 79.5 | 79.7 |

^*^ Limited to participants with ≥14 daytime and ≥6 nighttime measures

^†^ Diagnostic thresholds were ≥140 mmHg systolic or ≥90 mmHg diastolic for Clinic and ≥135 mmHg systolic or ≥85 mmHg diastolic for Home and Kiosk compared to 24-hour ABPM ≥130 mmHg diastolic or ≥80 diastolic mmHg

^‡^ Prevalence of hypertension based on 24-hour ABPM was 79.3% across all groups combined

Abbreviations: ABPM, ambulatory blood pressure monitoring; BP, blood pressure; CI, confidence interval; mmHg, millimeter of mercury; N, number for group; n, number for subgroup

**Appendix Table 5. Diagnostic Performance of Clinic, Home, and Kiosk BP Monitoring Compared**

**to Nighttime ABPM ^*,†^**

|  | **Clinic** | **Home** | **Kiosk** |
| --- | --- | --- | --- |
|  | N=141 | N=152 | N=138 |
| Nighttime mean ABPM ≥120/70 mmHg ^‡^, % | 83.7 | 79.0 | 81.9 |
| Sensitivity (95% CI) | 30.5 (22.9, 39.4) | 81.7 (73.7, 87.6) | 92.9 (86.5, 96.4) |
| Specificity (95% CI) | 82.6 (61.8, 93.3) | 65.6 (47.9, 79.8) | 28.0 (14.0, 48.2) |
| Positive Predictive Value (95% CI) | 90.0 (76.2, 96.2) | 90.0 (82.7, 94.3) | 85.4 (78.0, 90.6) |
| Negative Predictive Value (95% CI) | 18.8 (12.3, 27.6) | 48.8 (34.4, 63.4) | 46.7 (24.1, 70.7) |
| Positive Likelihood Ratio (95% CI) | 1.75 (0.69, 4.45) | 2.38 (1.46, 3.86) | 1.29 (1.01, 1.66) |
| Negative Likelihood Ratio (95% CI) | 0.84 (0.67, 1.05) | 0.28 (0.18, 0.44) | 0.25 (0.10, 0.63) |
| True Positive (Hypertension), n (%) | 36 (25.5) | 98 (64.5) | 105 (76.1) |
| True Negative (Normotensive), n (%) | 19 (13.5) | 21 (13.8) | 7 (5.1) |
| False Positive, n (%) | 4 (2.8) | 11 (7.2) | 18 (13.0) |
| False Negative (Missed Hypertension), n (%) | 82 (58.2) | 22 (14.5) | 8 (5.8) |
| Correctly Classified (%) | 39.0 | 78.3 | 81.2 |

^*^ Limited to participants with 6 or more nighttime measures (no requirement on number of daytime measures)

^†^ Diagnostic thresholds were ≥140/90 mmHg for Clinic and ≥135/85 for Home and Kiosk compared to nighttime ABPM ≥120 or ≥70 mmHg

^‡^ Prevalence of hypertension based on nighttime ABPM was 81.4% across all groups combined

^§^ Spearman correlation between diagnostic measurement and mean nighttime ABPM measurement

Abbreviations: ABPM, ambulatory blood pressure monitoring; BP, blood pressure; CI, confidence interval; N, number for group; n, number for subgroup

**Appendix Table 6. Diagnostic Performance of Clinic, Home, and Kiosk BP Monitoring Compared**

**to Mean Daytime ABPM using ACC/AHA Guidelines for Stage 1 Hypertension^*†^**

|  | **Clinic BP** | **Home BP** | **Kiosk BP** |
| --- | --- | --- | --- |
|  | N=142 | N=152 | N=140 |
| Prevalence of Hypertension Based on ABPM^‡^ | 86.6 | 85.5 | 86.4 |
| Sensitivity (95% CI) | 84.6 (77.0, 89.9) | 93.1 (87.2, 96.4) | 96.7 (91.5, 98.8) |
| Specificity (95% CI) | 36.8 (18.7, 59.7) | 36.4 (19.3, 57.7) | 15.8 (5.2, 39.2) |
| Positive Predictive Value (95% CI) | 89.7 (82.7, 94.0) | 89.6 (83.2, 93.8) | 88.0 (81.3, 92.5) |
| Negative Predictive Value (95% CI) | 26.9 (13.4, 46.7) | 47.1 (25.5, 69.7) | 42.9 (14.4, 77.0) |
| Positive Likelihood Ratio (95% CI) | 1.34 (0.94, 1.90) | 1.46 (1.06, 2.01) | 1.15 (0.94, 1.40) |
| Negative Likelihood Ratio (95% CI) | 0.42 (0.20, 0.86) | 0.19 (0.08, 0.44) | 0.21 (0.05, 0.86) |
| True Positive (Hypertension), n (%) | 104 (73.2) | 121 (79.6) | 117 (83.6) |
| True Negative (Normotensive), n (%) | 7 (4.9) | 8 (5.3) | 3 (2.1) |
| False Positive, n (%) | 12 (8.5) | 14 (9.2) | 16 (11.4) |
| False Negative (Missed Hypertension), n (%) | 19 (13.4) | 9 (5.9) | 4 (2.9) |
| Correctly Classified (%) | 78.2 | 84.9 | 85.7 |

^*^ Among individuals who had ≥1 diagnostic (Clinic, Home, Kiosk) measurement, and ≥14 daytime ABPM measurements.

^†^ Diagnostic thresholds were ≥130 mmHg systolic or ≥80 mmHg diastolic for Clinic, Home, Kiosk and ABPM

^‡^ Prevalence of hypertension based on mean daytime ABPM was 86.2% across all groups combined

^§^ Spearman correlation between diagnostic measurement and mean 24-hour ABPM measurement

Abbreviations: ABPM, ambulatory blood pressure monitoring; ACC/AHA, American College of Cardiology/American Heart Association; BP, blood pressure; CI, confidence interval; mmHg, millimeter of mercury; N, number for group; n, number for subgroup

**Appendix Table 7. Diagnostic Performance of Clinic, Home, and Kiosk BP Compared to Daytime ABPM and Different BP Thresholds^*†^**

| Diagnostic BP | Thresholds mmHg | Above threshold  n (%) | Sensitivity  (95% CI) | Specificity  (95% CI) | PPV  (95% CI) | NPV  (95% CI) | Correctly Classified (%) |
| --- | --- | --- | --- | --- | --- | --- | --- |
| Clinic | 120/75 | 132 (93.0) | 93.2 (86.4, 96.7) | 7.7 (2.5, 21.3) | 72.7 (64.5, 79.6) | 30.0 (10.0, 62.4) | 69.7 |
| N=142 | 130/80 | 116 (81.7) | 86.4 (78.3, 91.8) | 30.8 (18.4, 46.7) | 76.7 (68.2, 83.5) | 46.2 (28.4, 65.0) | 71.1 |
|  | 135/85 | 82 (57.8) | 64.1 (54.4, 72.7) | 59.0 (43.2, 73.1) | 80.5 (70.5, 87.7) | 38.3 (27.0, 51.1) | 62.7 |
|  | 140/90 | 40 (28.2) | 31.1 (22.9, 40.6) | 79.5 (64.0, 89.4) | 80.0 (64.8, 89.7) | 30.4 (22.3, 40.0) | 44.4 |
|  | 150/95 | 17 (12.0) | 10.7 (6.0, 18.3) | 84.6 (69.7, 92.9) | 64.7 (40.4, 83.2) | 26.4 (19.4, 34.8) | 31.0 |
|  | 160/100 | 7 (4.9) | 4.9 (2.0, 11.1) | 94.9 (81.7, 98.7) | 71.4 (32.7, 92.8) | 27.4 (20.6, 35.5) | 29.6 |
| Home | 120/75 | 149 (98.0) | 100 (‡) | 6.7 (2.2, 18.7) | 71.8 (64.1, 78.4) | 100 (§) | 72.4 |
| N=152 | 130/80 | 135 (88.8) | 95.3 (89.3, 98.0) | 26.7 (15.8, 41.3) | 75.6 (67.6, 82.1) | 70.6 (45.8, 87.2) | 75.0 |
|  | 135/85 | 109 (71.7) | 82.2 (73.8, 88.4) | 53.3 (38.9, 67.2) | 80.7 (72.2, 87.1) | 55.8 (40.9, 69.8) | 73.7 |
|  | 140/90 | 73 (48.0) | 57.0 (47.5, 66.0) | 73.3 (58.7, 84.2) | 83.6 (73.2, 90.4) | 41.8 (31.4, 52.9) | 61.8 |
|  | 150/95 | 30 (19.7) | 25.2 (17.9, 34.3) | 93.3 (81.3, 97.8) | 90.0 (73.2, 96.7) | 34.4 (26.5, 43.3) | 45.4 |
|  | 160/100 | 10 (6.6) | 9.3 (5.1, 26.9) | 100 (^†^) | 100 (^‡^) | 31.7 (24.6, 39.8) | 36.2 |
| Kiosk | 120/75 | 139 (99.3) | 100 (‡) | 2.6 (0.4, 16.1) | 72.7 (64.7, 79.4) | 100 (§) | 72.9 |
| N=140 | 130/80 | 133 (95.0) | 99.0 (93.3, 99.9) | 15.4 (7.1, 30.3) | 75.2 (67.2, 81.8) | 85.7 (41.9, 98.0) | 75.7 |
|  | 135/85 | 125 (89.3) | 96.0 (89.9, 98.5) | 28.2 (16.4, 44.1) | 77.6 (69.5, 84.1) | 73.3 (46.7, 89.6) | 77.1 |
|  | 140/90 | 110 (78.6) | 85.1 (76.8, 90.8) | 38.5 (24.7, 54.4) | 78.2 (69.5, 84.9) | 50.0 (32.8, 67.2) | 72.1 |
|  | 150/95 | 75 (53.6) | 61.4 (51.6, 70.4) | 66.7 (50.7, 79.6) | 82.7 (72.4, 89.7) | 40.0 (28.9, 52.3) | 62.9 |
|  | 160/100 | 41 (29.3) | 35.6 (26.9, 45.4) | 87.2 (72.7, 94.6) | 87.8 (73.9, 94.8) | 34.3 (25.6, 44.2) | 50.0 |

^*^Among individuals who had ≥1 diagnostic (Clinic, Home, Kiosk) measurement, and ≥14 daytime ABPM measurements.

†Reference standard: ABPM mean daytime ≥135 mmHg systolic or ≥85 mmHg diastolic

‡Not estimable, no variability in diagnostic test result, given ABPM result

§Not estimable, no variability in ABPM result, given diagnostic test result

Abbreviations: ABPM, ambulatory blood pressure monitoring; BP, blood pressure; CI, confidence interval; mmHg, millimeter of mercury; NPV, negative predictive value; PPV, positive predictive value; N, number for group; n, number for subgroup

**Appendix Table 8.a. Clinic Hypertension Diagnostic Performance by Patient Characteristics^*^**

|  | N | Prevalence  Hypertension by ABPM^a^(%) | Mean Differences in Systolic BP^†^ (95% CI) | Mean Differences in Diastolic BP^†^  (95% CI) | Sensitivity, %  (95% CI) | Specificity, %  (95% CI) | Correctly  Classified (%) |
| --- | --- | --- | --- | --- | --- | --- | --- |
| Overall | 142 | 72.5 | -4.7 (-7.3, -2.2) | -7.2 (-8.8, -5.5) | 31.1 (22.9, 40.6) | 79.5 (64.0, 89.4) | 44.4 |
| Age |  |  |  |  |  |  |  |
| <60 years | 62 | 79.0 | -5.6 (-9.7, -1.5) | -8.7 (-11.2, -6.1) | 20.4 (11.3, 33.9) | 84.6 (54.9, 96.1) | 33.9 |
| ≥60 years | 80 | 67.5 | -4.1 (-7.5, -0.7) | -6.0 (-8.1, -4.0) | 40.7 (28.5, 54.2) | 76.9 (57.2, 89.2) | 52.5 |
| Sex |  |  |  |  |  |  |  |
| Male | 69 | 76.8 | -5.3 (-8.9, -1.6) | -8.3 (-10.6, -6.0) | 30.1 (19.4, 43.7) | 87.5 (61.4, 96.9) | 43.5 |
| Female | 73 | 68.5 | -4.3 (-7.9, -0.7) | -5.8 (-8.1, -3.5) | 32.0 (20.6, 46.0) | 73.9 (52.7, 87.8) | 45.2 |
| Baseline BP |  |  |  |  |  |  |  |
| <150 mmHg | 73 | 68.5 | -5.7 (-8.6, -2.8) | -7.2 (-9.3, -5.0) | 26.0 (15.7, 39.8) | 95.6 (74.8, 99.3) | 48.0 |
| ≥150 mmHg | 69 | 76.8 | -3.8 (-8.4, 0.9) | -7.1 (-9.6, -4.6) | 35.8 (24.2, 49.5) | 56.3 (32.4, 77.8) | 40.6 |
| BMI^‡^ |  |  |  |  |  |  |  |
| <30 kg/m^2^ | 68 | 64.7 | -3.7 (-7.3, -0.2) | -8.2 (-10.5, -5.9) | 29.5 (18.0, 44.5) | 87.5 (67.6, 95.9) | 50.0 |
| ≥30 kg/m^2^ | 73 | 79.5 | -5.6 (-9.4, -1.9) | -5.6 (-7.7, -3.4) | 32.8 (22.0, 45.7) | 66.7 (40.6, 85.4) | 39.7 |
| Arm Size |  |  |  |  |  |  |  |
| <33 cm | 78 | 64.1 | -2.5 (-5.8, 0.8) | -6.9 (-9.1, -4.7) | 34.0 (22.3, 48.0) | 89.3 (71.6, 96.5) | 53.9 |
| ≥33 cm | 64 | 82.8 | -7.5 (-12.0, -3.0) | -7.3 (-9.9, -4.7) | 28.3 (17.8, 41.8) | 54.5 (26.8, 79.7) | 32.8 |
| 10-year CVD risk^‡,§^ |  |  |  |  |  |  |  |
| <15% | 61 | 73.8 | -6.3 (-10.1, -2.4) | -9.1 (-11.7, -6.4) | 22.2 (12.4, 36.6) | 100 (^II^) | 42.6 |
| >15% | 79 | 72.2 | -4.2 (-7.8, -0.6) | -6.2 (-8.3, -4.0) | 36.8 (25.4, 50.0) | 68.2 (46.6, 84.0) | 45.6 |
| Race |  |  |  |  |  |  |  |
| White | 111 | 74.8 | -4.1 (-6.9, -1.3) | -6.5 (-8.2, -4.7) | 33.7 (24.4, 4.5) | 75.0 (56.1, 87.6) | 44.1 |
| Black | 11 | 63.6 | -10.4 (-18.4, -2.3) | -13.2 (-19.2, 7.2) | 14.3 (2.0, 58.1) | 100 (^II^) | 45.5 |
| Other | 20 | 65.0 | -5.2 (-13.2, 2.8) | -8.3 (-12.6, -3.9) | 23.1 (7.6, 52.2) | 85.7 (41.9, 98.0) | 45.0 |
| Education^‡^ |  |  |  |  |  |  |  |
| <College grad | 52 | 69.2 | -2.1 (-6.3, 2.1) | -7.2 (-10.0, -4.3) | 36.1 (22.3, 52.7) | 62.5 (37.7, 82.1) | 44.2 |
| College grad | 41 | 78.1 | -5.1 (-9.3, -0.9) | -7.7 (-10.7, -4.6) | 31.3 (17.7, 49.0) | 100 (^II^) | 46.3 |
| Post grad | 45 | 73.3 | -7.4 (-12.2, -2.5) | -6.7 (-9.1, -4.3) | 27.3 (14.8, 44.7) | 83.3 (52.3, 95.8) | 42.2 |

^*^ Using mean daytime ambulatory BP ≥135 mmHg systolic or ≥85 mmHg diastolic as the reference standard

^†^ Linear regression models adjusted for age, sex, body mass index, education, and baseline systolic and diastolic BP (from screening visit), estimated with generalized estimating equations with robust (sandwich) variance estimation

^‡^ Missing values: BMI (n=1), CVD risk (n=2), education (n=4)

^§^ Moderate-to-high risk for CVD defined as prevalent CVD, age ≥75, statin prescribed in the past 12 months, or ≥15% 10-year risk using Framingham 10-year risk equations. Framingham risk defined using participants’ most recent cholesterol prior to enrollment. When cholesterol data were unavailable, BMI was used to calculate risk

^II^ Not estimable, no variability in diagnostic test result given ABPM result

Abbreviations: ABPM, ambulatory blood pressure monitoring; BP, blood pressure; BMI, body mass index; CVD, cardiovascular disease; CI, confidence interval; mmHg, millimeter of mercury

**Appendix Table 8.b. Home Hypertension Diagnostic Performance by Patient Characteristics**^*^

|  | N | Prevalence  Hypertension by ABPM^a^ (%) | Mean Differences in Systolic BP^†^  (95% CI) | Mean Differences in Diastolic BP^†^  (95% CI) | Sensitivity, % (95% CI) | Specificity, %  (95% CI) | Correctly  Classified (%) |
| --- | --- | --- | --- | --- | --- | --- | --- |
| Overall | 152 | 70.4 | -0.1 (-1.6, 1.5) | -0.4 (-1.4, 0.7) | 82.2 (73.8, 88.4) | 53.3 (38.9, 67.2) | 73.7 |
| Age |  |  |  |  |  |  |  |
| <60 years | 70 | 74.3 | -2.8 (-5.3, -0.2) | -0.9 (-2.6, 0.9) | 80.8 (67.8, 89.3) | 44.4 (24.0, 67.0) | 71.4 |
| ≥60 years | 82 | 67.1 | 2.3 (-0.1, 4.6) | 0.1 (-1.4, 1.5) | 83.6 (71.4, 91.3) | 59.3 (40.3, 75.8) | 75.6 |
| Sex |  |  |  |  |  |  |  |
| Male | 76 | 77.6 | 0.4 (-1.6, 2.5) | -3.5 (-4.8, -2.2) | 83.1 (71.3, 90.6) | 58.8 (35.2, 78.9) | 77.6 |
| Female | 76 | 63.2 | -0.5 (-2.8, 1.9) | 3.0 (1.4, 4.6) | 81.3 (67.7, 89.9) | 50.0 (32.3, 67.7) | 69.7 |
| Baseline BP |  |  |  |  |  |  |  |
| <150 mmHg | 80 | 63.8 | -0.3 (-2.3, 1.7) | 0.3 (-1.1, 1.7) | 84.3 (71.6, 92.0) | 65.5 (46.9, 80.3) | 77.5 |
| ≥150 mmHg | 72 | 77.8 | 0.2 (-2.3, 2.7) | -1.1 (-2.8, 0.5) | 80.4 (67.9, 88.8) | 31.3 (13.6, 56.7) | 69.4 |
| BMI^‡^ |  |  |  |  |  |  |  |
| <30 kg/m^2^ | 92 | 71.7 | -1.6 (-3.5, 0.4) | -2.4 (-3.8, -1.1) | 75.8 (64.0, 84.6) | 65.4 (45.7, 80.9) | 72.8 |
| ≥30 kg/m^2^ | 59 | 67.8 | 2.3 (-0.2, 4.8) | 3.2 (1.4, 5.0) | 92.5 (79.2, 97.6) | 36.8 (18.7, 59.7) | 74.6 |
| Arm Size |  |  |  |  |  |  |  |
| <33 cm | 86 | 67.4 | -1.3 (-3.6, 1.0) | -1.1 (-2.7, 0.4) | 74.1 (61.4, 83.8) | 67.9 (48.9, 82.4) | 72.1 |
| ≥33 cm | 66 | 74.2 | 1.5 (-1.0, 4.0) | 0.7 (-1.1, 2.5) | 91.8 (80.2, 96.9) | 29.4 (12.8, 54.2) | 75.8 |
| 10-year CVD Risk^‡,§^ |  |  |  |  |  |  |  |
| <15% | 68 | 72.1 | -2.2 (-5.0, 0.5) | -0.6 (-2.5, 1.3) | 79.6 (66.1, 88.7) | 47.4 (26.8, 68.9) | 70.6 |
| >15% | 82 | 68.3 | 1.7 (-0.7, 4.2) | -0.1 (-1.7, 1.4) | 85.7 (73.9, 92.7) | 57.7 (38.5, 74.8) | 76.8 |
| Race |  |  |  |  |  |  |  |
| White | 123 | 70.7 | -0.2 (-1.9, 1.6) | -0.9 (-2.0, 0.3) | 81.6 (72.1, 88.4) | 58.3 (41.9, 73.1) | 74.8 |
| Black | 11 | 63.6 | 4.0 (-33.2, 11.2) | 3.4 (-0.6, 7.4) | 100 (^II^) | 0 (^II^) | 63.6 |
| Other | 18 | 72.2 | -1.9 (-5.7, 1.9) | 1.0 (-2.4, 4.4) | 76.9 (47.8, 92.4) | 60.0 (20.0, 90.0) | 72.2 |
| Education^‡^ |  |  |  |  |  |  |  |
| <College grad | 53 | 62.3 | 0.5 (-2.1, 3.0) | -1.2 (-3.3, 0.9) | 75.8 (58.5, 87.4) | 45.0 (25.3, 66.4) | 64.2 |
| College grad | 46 | 67.4 | -1.6 (-4.3, 1.1) | 0.0 (-1.5, 1.5) | 87.1 (70.3, 95.1) | 66.7 (40.6, 85.4) | 80.4 |
| Post grad | 51 | 82.4 | 0.6 (-2.2, 3.5) | 0.2 (-1.5, 1.8) | 83.3 (69.0, 91.8) | 55.6 (25.1, 82.3) | 78.4 |

^*^ Using mean daytime ambulatory BP ≥135 mmHg systolic or ≥85 mmHg diastolic as the reference standard

^†^ Linear regression models adjusted for age, sex, body mass index, education, and baseline systolic and diastolic BP (from screening visit), estimated with generalized estimating equations with robust (sandwich) variance estimation

^‡^ Missing values: BMI (n=1), CVD risk (n=2), education (n=2)

^§^ Moderate-to-high risk for CVD defined as prevalent CVD, age ≥75, statin prescribed in the past 12 months, or ≥15% 10-year risk using Framingham 10-year risk equations. Framingham risk defined using participants’ most recent cholesterol prior to enrollment. When cholesterol data were unavailable, BMI was used to calculate risk

^II^ Not estimable, no variability in diagnostic test result given ABPM result

Abbreviations: ABPM, ambulatory blood pressure monitoring; BP, blood pressure; BMI, body mass index; CVD, cardiovascular disease; CI, confidence interval; mmHg, millimeter of mercury

**Appendix Table 8.c. Kiosk Hypertension Diagnostic Performance by Patient Characteristics** ^*^

|  | N | Prevalence  Hypertension by ABPM (%) | Mean Differences in Systolic BP  (95% CI)^†^ | Mean Differences in Diastolic BP  (95% CI) ^†^ | Sensitivity, % (95% CI) | Specificity, %  (95% CI) | Correctly  Classified (%) |
| --- | --- | --- | --- | --- | --- | --- | --- |
| Overall | 140 | 72.1 | 9.5 (7.5, 11.6) | 5.0 (3.8, 6.2) | 96.0 (90.0, 98.5) | 28.2 (16.4, 44.1) | 77.1 |
| Age |  |  |  |  |  |  |  |
| <60 years | 66 | 80.3 | 8.8 (5.9, 11.8) | 6.0 (4.2, 7.8) | 96.2 (86.1, 99.1) | 20.1 (7.6, 52.2) | 81.8 |
| ≥60 years | 74 | 64.9 | 10.1 (7.2, 13.0) | 4.0 (2.3, 5.7) | 95.8 (84.8, 99.0) | 30.8 (16.2, 50.5) | 73.0 |
| Sex |  |  |  |  |  |  |  |
| Male | 77 | 76.6 | 8.6 (6.8, 10.4) | 3.8 (2.3, 5.2) | 94.9 (85.4, 98.4) | 27.8 (12.1, 51.9) | 79.2 |
| Female | 63 | 66.7 | 10.6 (6.5, 14.7) | 6.1 (4.1, 8.2) | 97.6 (84.9, 99.7) | 28.6 (13.4, 50.8) | 74.6 |
| Baseline BP |  |  |  |  |  |  |  |
| <150 mmHg | 74 | 67.6 | 7.4 (5.2, 9.6) | 5.7 (4.3, 7.2) | 94.0 (83.0, 98.1) | 33.3 (17.6, 53.9) | 74.3 |
| ≥150 mmHg | 66 | 77.3 | 12.0 (8.4, 15.6) | 4.1 (2.1, 6.1) | 98.0 (87.4, 99.7) | 20.0 (6.6, 47.0) | 80.3 |
| BMI^‡^ |  |  |  |  |  |  |  |
| <30 kg/m^2^ | 91 | 68.1 | 9.5 (6.9, 12.1) | 3.7 (2.3, 5.2) | 95.2 (86.0, 98.4) | 37.9 (22.4, 56.4) | 76.9 |
| ≥30 kg/m^2^ | 48 | 79.2 | 9.5 (6.1, 12.9) | 6.1 (3.8, 8.4) | 97.4 (83.5, 99.6) | 0 (^§^) | 77.1 |
| Arm Size |  |  |  |  |  |  |  |
| <33 cm | 83 | 72.3 | 8.6 (5.6, 11.6) | 4.2 (2.3, 6.1) | 93.3 (83.5, 97.5) | 34.8 (18.4, 55.7) | 77.1 |
| ≥33 cm | 57 | 71.9 | 10.9 (8.5, 13.4) | 5.9 (4.4, 7.5) | 100 (^§^) | 18.8 (6.2, 44.7) | 77.2 |
| 10-year CVD Risk ^‡,II^ |  |  |  |  |  |  |  |
| <15% | 65 | 72.3 | 9.9 (6.4, 13.4) | 5.2 (3.4, 7.0) | 97.9 (86.4, 99.7) | 16.7 (5.5, 40.9) | 75.4 |
| >15% | 73 | 72.6 | 9.2 (6.3, 12.1) | 4.7 (2.8, 6.6) | 94.3 (83.9, 98.2) | 35.0 (17.7, 57.4) | 78.1 |
| Race |  |  |  |  |  |  |  |
| White | 113 | 69.9 | 8.9 (6.9, 10.8) | 4.4 (3.1, 5.7) | 96.2 (88.9, 98.8) | 29.4 (16.6, 46.6) | 76.1 |
| Black | 6 | 83.3 | 22.8 (10.6, 35.1) | 11.8 (7.2, 16.5) | 100 (^II^) | --^¶^ | 83.3 |
| Other | 21 | 81.0 | 8.7 (1.6, 15.9) | 6.2 (2.6, 9.9) | 94.1 (68.0, 99.2) | 25.0 (3.3, 76.2) | 81.0 |
| Education^‡^ |  |  |  |  |  |  |  |
| <College grad | 53 | 62.3 | 13.1 (9.2, 17.1) | 7.6 (5.5, 9.8) | 90.9 (75.3, 97.0) | 15.0 (4.9, 37.6) | 62.3 |
| College grad | 40 | 85.0 | 7.0 (3.3, 10.8) | 4.6 (2.6, 6.5) | 97.1 (81.9, 99.6) | 50.0 (16.8, 83.2) | 90.0 |
| Post grad | 45 | 71.1 | 7.7 (5.0, 10.4) | 2.4 (0.5, 4.3) | 100 (^II^) | 38.5 (17.0, 65.6) | 82.2 |

^*^ Using mean daytime ambulatory BP ≥135 mmHg systolic or ≥85 mmHg diastolic as the reference standard

^†^ Linear regression models adjusted for age, sex, body mass index, education, and baseline systolic and diastolic BP (from screening visit), estimated with generalized estimating equations with robust (sandwich) variance estimation

^‡^ Missing values: BMI (n=1), CVD risk (n=2), education (n=2)

^§^ Not estimable, no variability in diagnostic test result given ABPM result

^II^ Moderate-to-high risk for CVD defined as prevalent CVD, age ≥75, statin prescribed in the past 12 months, or ≥15% 10-year risk using Framingham 10-year risk equations. Framingham risk defined using participants’ most recent cholesterol prior to enrollment. When cholesterol data were unavailable, BMI was used to calculate risk

^¶^ No individuals without hypertension by ABPM

Abbreviations: ABPM, ambulatory blood pressure monitoring; BP, blood pressure; BMI, body mass index; CVD, cardiovascular disease; CI, confidence interval; mmHg, millimeter of mercury

**Appendix Figure 1: Bland-Altman Plots Comparing the Mean Versus the Difference Between the Diagnostic BP Measure (Clinic, Home, and Kiosk) and Mean Daytime Ambulatory BP**. 95% limits of agreement were defined as the unadjusted mean difference between daytime ABPM and diagnostic BP ± 1.96 standard deviation of the difference. Dotted gray line at 0 shows the reference line where mean ABPM BP is equal to the mean diagnostic BP. This line represents the case where ABPM and diagnostic BP are equal. Green dots represent observed data for each participant. Solid blue line represents the group mean difference between the diagnostic regimen and ABPM reference standard. Dashed red lines represent the 95% limits of agreement for the mean difference between the diagnostic regimen and the ABPM reference standard. Bias is the unadjusted mean difference between ABPM and diagnostic BP measures. Abbreviations: BP, blood pressure; ABPM, ambulatory BP monitoring; mmHg, millimeters of mercury

**Appendix Figure 2.** Receiver Operator Characteristics (ROC) Clinic, Home, and Kiosk BP Based on the Reference Standard: Mean Daytime ABPM Thresholds of Systolic 135 mmHg and Diastolic 85 mmHg


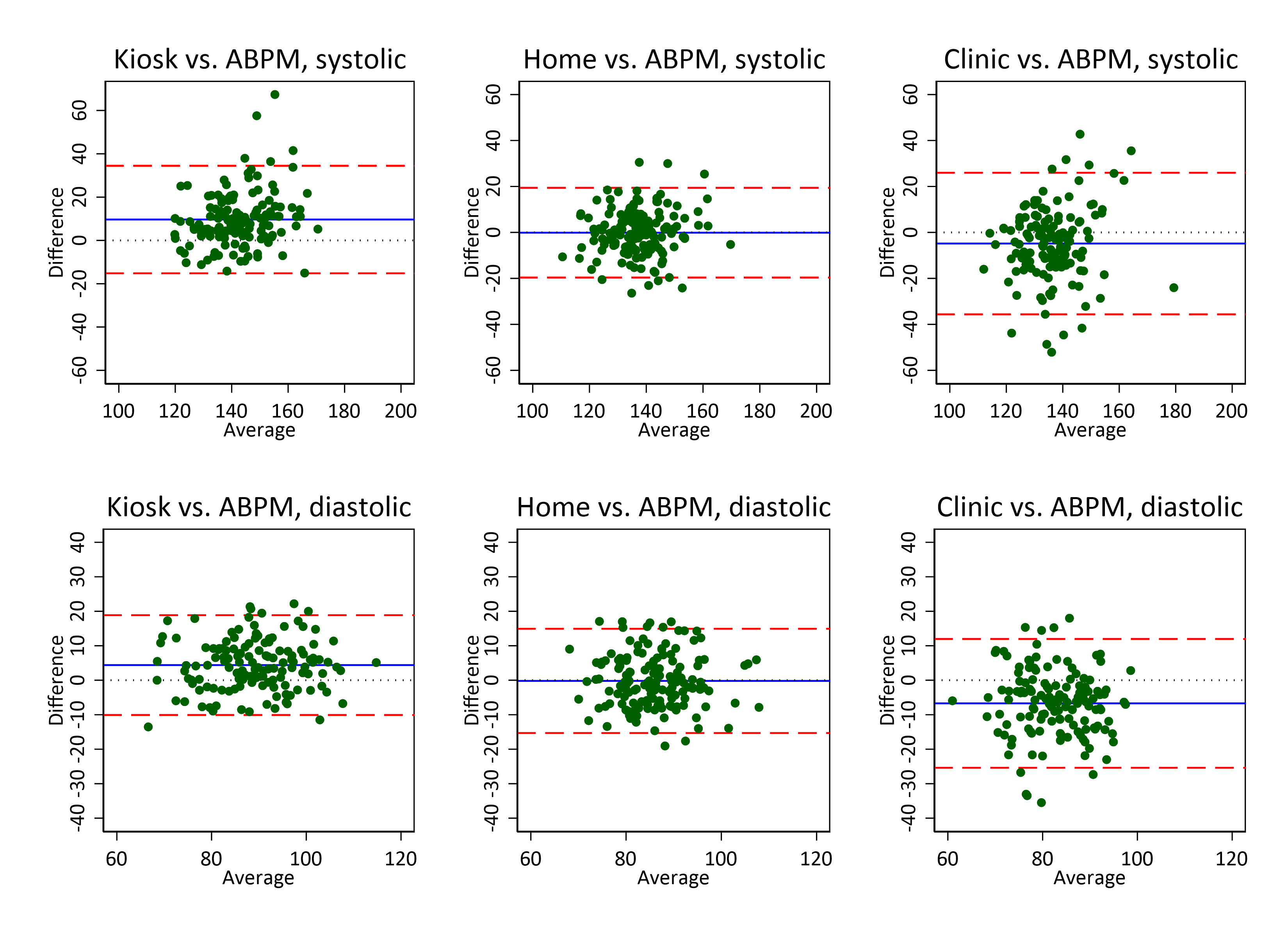


| **Appendix Figure 2.** Receiver Operator Characteristics (ROC) Clinic, Home, and Kiosk BP Based on the Reference Standard: Daytime ABPM Thresholds of Systolic 135 mmHg and Diastolic 85 mmHg | | | | |
| --- | --- | --- | --- | --- |
|  | | |  | |
| Area-under-the-curve (AUC) for the ROC curve | Clinic | Home | | Kiosk |
|  | AUC (95% CI) | AUC (95% CI) | | AUC (95% CI) |
| Systolic | 0.64 (0.54, 0.71) | 0.77 (0.69, 0.84) | | 0.75 (0.70, 0.84) |
| Diastolic | 0.75 (0.67, 0.83) | 0.75 (0.67, 0.92) | | 0.86 (0.79, 0.92) |

ABPM ambulatory blood pressure monitoring; AUC, area under the curve; BP, blood pressure; CI, confidence interval; ROC, receiver operating characteristics
